# Experience of bipolar androgen therapy (BAT) in Argentinian oncology centres

**DOI:** 10.3332/ecancer.2022.1480

**Published:** 2022-12-02

**Authors:** Martín Zarbá, Martin Angel, Federico Losco, Juan José Zarbá, Juan Carlos Pupilli, Matías Rodrigo Chacon, Juan Pablo Sade

**Affiliations:** 1 FUCA, Instituto Alexander Fleming, CABA C1426ANZ, Argentina; 2 Genitourinary Tumors Department, Instituto Alexander Fleming, CABA C1426ANZ, Argentina; 3 Oncology Department, Hospital Zenon Santillan, San Miguel de Tucuman T4000IAK, Argentina; 4 Genitourinary Tumors Department, Sanatorio Británico Rosario, Santa Fé S2000ANZ, Argentina; 5 Oncology Department, Instituto Alexander Fleming, CABA C1426ANZ, Argentina; † These authors have contributed equally to this work.; a https://orcid.org/0000-0003-3642-4035; b https://orcid.org/0000-0002-1463-8887; c https://orcid.org/0000-0001-5084-3012; d https://orcid.org/0000-0003-1013-3993; e https://orcid.org/0000-0001-6872-4185; f https://orcid.org/0000-0001-9312-5280

**Keywords:** androgen deprivation, castration resistance, supraphysiological testosterone

## Abstract

**Background:**

Previous studies with bipolar androgen therapy (BAT) have shown clinical activity in metastatic Castration Resistant Prostate Cancer (mCRPC) as well as the potential to re-sensitise prostate cancer cells to prior androgen receptor-targeted agents. None of these studies had tested BAT after chemotherapy. In this study, we gathered real-world evidence from three centres in Argentina where BAT is being used in castration-resistant prostate cancer (CRPC), not only prior to chemotherapy but also after several lines of treatment.

**Materials and methods:**

This retro-prospective nonrandomised multicentre cohort study included patients with mCRPC, who received BAT in different scenarios defined by the treating physician at three centres in Argentina.

**Results:**

A total of 21 asymptomatic patients with mCRPC were included. There was a median of two lines before BAT, with nine patients (42.8%) receiving three or more lines, and 13 patients (61.9%) receiving chemotherapy previously. Previous lines included next-generation hormonal agents (NHA) in 100% (abiraterone 33.3% and enzalutamide 71.4%), chemotherapy in 61.9%, Radium-223 in 47.6% and others in 4.8%. The progression free survival (PFS) after BAT was 3.5 months (95% CI: 3.06–7.97). PSA50 response rate (RR) was 28.5% and the overall RR was 14.3%. Of the 17 patients who had disease progression, 9 had a rechallenge to NHA, achieving a 55% RR, 6 received other treatment (chemotherapy in 5 and ^177^Lu-PSMA in 1) with a 66% RR and 2 best supportive care. The PFS2, calculated after the initiation of BAT in the 15 patients who received further therapy, was 7.93 months (95% CI: 6.73–NR). Treatment was overall well tolerated, with only two patients requiring hospitalisation and treatment interruption due to worsening pain.

**Conclusion:**

To the authors’ knowledge, this is the first publication of BAT in later lines of therapy in mCRPC. BAT showed clinical activity in this scenario. Our data supports that BAT may play a role in CRPC re-sensitisation after multiple treatment lines.

## Introduction

Throughout the entire course of the disease, prostate cancer cells are dependent on the androgen receptor (AR) signalling [1]. Therefore, androgen deprivation therapy (ADT) is a highly effective treatment for this illness and has remained as the backbone of care since Huggins and Hodges [2] research in the 40s. However, ADT is not curative, and practically all patients will eventually experience disease progression to a castration-resistant prostate cancer (CRPC) [3]. Even then, ADT should not be suspended, as prostate cancer cells are still addicted to AR signalling and there is a survival benefit with continued testicular androgen suppression [4].

Resistance to ADT is explained primarily due to molecular adaptations within the AR axis, such as AR overexpression, gene amplification, gain of function point mutations and splice variants of the receptor that lead to transcriptional activity even with the loss of its ligand-binding domain. The result of these adaptive changes is an increase in AR that maintains its activity despite castration levels of testosterone [5, 6]. As the Cabazitaxel versus Abiraterone or Enzalutamide in Metastatic Prostate Cancer (CARD) trial showed, in agreement with other studies, a second androgen signalling targeted inhibitor shows poor outcomes, probably due to the fact that these agents target the same pathway and share common mechanisms of resistance [7–11].

Even though these AR changes produce resistance to ADT, they also produce a liability that can be exploited therapeutically. Experimental data suggested that rapid cycling between supraphysiologic (>1,500 ng/dL) and castrate (<50 ng/dL) testosterone levels (bipolar androgen therapy (BAT)) may re-sensitise CRPC to further androgen-directed therapies [12, 13].

Even though BAT is a promising strategy for the treatment of CRPC and there is growing evidence to support its use, its indication is still not clear, mainly because different trials have tested it in different clinical scenarios, and, to this date, it is still absent in major guidelines [14, 15]. Real-life evidence is scarce and there are no reports found in the literature of BAT in patients with CRPC after chemotherapy [16].

In this study, we aimed to report real-world evidence from different cancer centres in Argentina where BAT is being used in CRPC, not only prior to chemotherapy but also after several lines of treatment.

## Materials and methods

### Study population and treatment characteristics

This retro-prospective nonrandomised multicentre cohort study included patients seen between 1 January 2017, and 28 February 2022, with a histologically confirmed diagnosis of prostate cancer, in the scenario of castration resistance, who received BAT at any point of the evolution of the disease at three cancer centres in Argentina. Cancer centres are geographically distributed among the country and also have different insurance coverage (private and public patients).

BAT consisted of monthly intramuscular testosterone cypionate (500 mg every 4 weeks) in addition to ongoing luteinising hormone-releasing hormone agonist therapy. Patients were treated until disease progression or unacceptable toxicity. Demographic and clinicopathological characteristics, including age, Eastern Cooperative Oncology Group performance status (ECOG-PS), Gleason score, prostate specific antigen (PSA) levels, comorbidities and previous treatments were collected from medical charts and entered into a predefined centralised database. Safety information was also retrieved, and subsequent treatment strategies, responses, adverse events and discontinuation were also documented.

Disease progression dates and treatment responses were collected from medical charts. Treatment response was evaluated by the investigator-assessed 50% declines in PSA concentration from baseline (PSA50) and by computed tomography or nuclear medicine bone scans in accordance with Prostate Cancer Working Group 3.

Progression-free survival 1 (PFS1) is defined as the time from the first injection of testosterone cypionate to disease progression or death from any cause and PFS2 as the time from the first injection of testosterone cypionate to second objective disease progression or death from any cause, whichever first.

All the patients granted written informed consent to collect medical information from institutional records.

Adverse events were graded as they were registered by the treating physician and were graded according to the National Cancer Institute Common Terminology Criteria for Adverse Events (version 5.0) when this information was missing.

### Statistical analysis

Data were summarised as frequencies and percentages for categorical variables and as medians, ranges and interquartile ranges for continuous variables. Data were censored at the last follow-up if the patient was alive. Survival curves were generated using the Kaplan–Meier method. All statistical analyses were performed using Statistical Package for the Social Sciences software version 23.0 (SPSS, Inc., Armonk, NY, USA).

## Results

In this multicentre retro-prospective study, a total of 21 patients with mCRPC were included from three Argentinian cancer centres. All patients received BAT in the castrate-resistant scenario at any line of treatment, independent of previous lines. The median age was 75 years (range: 64–83), most patients had an ECOG-PS of 0–1 (90%), although one patient had an ECOG-PS of 3. None of the patients received BAT as first-line treatment of CRPC, with a median of two previous lines, nine patients (42.8%) receiving more than two and one patient (4.7%) receiving five lines of treatments previously. Previous lines included next-generation hormonal agents (NHA) in 100% (abiraterone 33.3% and enzalutamide 71.4%), chemotherapy (docetaxel or cabazitaxel) in 61.9%, Radium-223 in 47.6% and poly (ADP-ribose) polymerase inhibitors in 4.7% (in context of a clinical trial). Baseline characteristics of all the patients are summarised in Table 1.

The cases included are summarised in Table 2.

**Figure 1.**
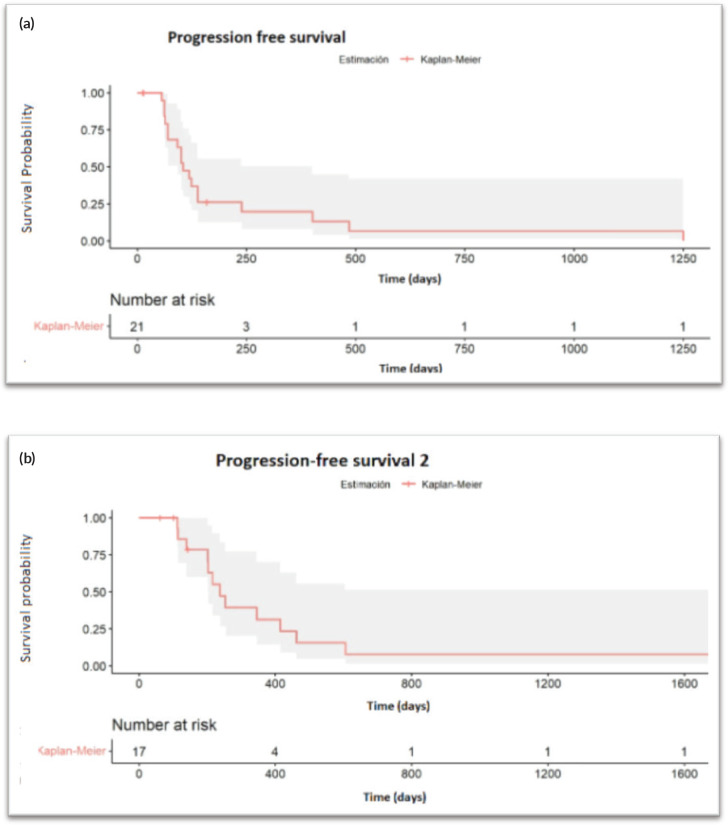
(a): Progression free survival. (b): Progression free survival 2.

**Figure 2.**
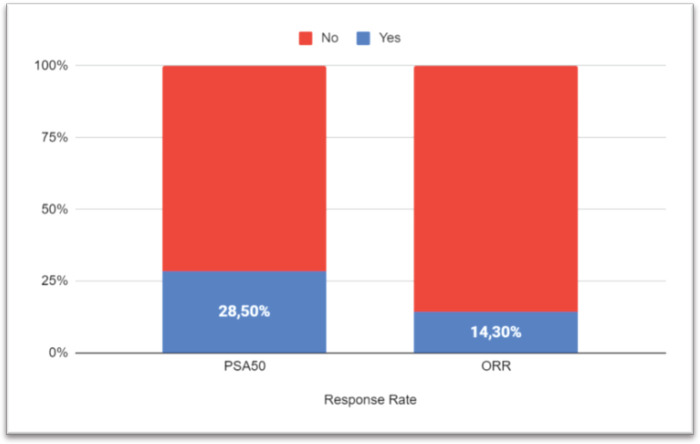
Response rate. PSA, Prostatic specific antigen; ORR, Overall response rate.

After a median follow-up of 6.7 months (95% CI: 3.7–11.5), 17 progression events occurred. The median PFS1 was 3.5 months (95% CI: 3.06–7.97) (Figure 1a). PSA50 response rate (RR) was 28.5% and the overall RR was 14.3% (Figure 2). Of the 17 patients who progressed to BAT, 15 (88.2%) received another line of treatment, and 9 of them (52.9%) had a rechallenge to prior NHA. Response, either clinical or biochemical, was achieved in 5 (55%) of patients who were retreated with NHA. Of the five patients who responded, four were rechallenged with enzalutamide, and one with abiraterone; three after one line and the other two after three lines of treatment. The median PFS2, in the 15 patients who received further therapy, was 7.93 months (95% CI: 6.73–NR) (Figure 1b).

Treatment was overall well tolerated. Most patients were asymptomatic at time of BAT initiation, only two patients presented with grade 1 bone pain at baseline. Main adverse events were grade 3 (worsening of pain) pain in two patients (9.5%) (requiring hospitalisation and treatment interruption), grade 1 headache in one patient (4.75%) and grade 1 lower limb oedema in one patient (4.75%). There were no grade 4 or 5 adverse events.

## Discussion

Several trials and a systematic review have tried to demonstrate the efficacy of BAT in patients with mCRPC, especially its effects in the re-sensitisation to hormonal therapy, but none of these trials tested BAT in heavily pretreated patients or in patients with previous use of chemotherapy. A randomised multicentre phase II trial comparing BAT versus enzalutamide in 195 men with CRPC progressed to abiraterone showed similar clinical/radiographic PFS and PSA50 in both arms, with longer biochemical PFS with BAT. Interestingly, the PSA-PFS for enzalutamide increased from 3.8 months after abiraterone to 10.9 months after BAT, and PFS2 with BAT followed by enzalutamide was significantly longer than with enzalutamide followed by BAT, proving the hypothesis that BAT can re-sensitise CRPC to subsequent antiandrogen therapy [17].

Another phase II multi cohort trial evaluated BAT in men with metastatic and non-metastatic CRPC. Two cohorts evaluated whether BAT could restore sensitivity to abiraterone and enzalutamide by treating patients who previously failed to one of these therapies with BAT and then subsequent retreatment with abiraterone or enzalutamide. In the enzalutamide cohort, a 50% decrease in PSA (PSA50) was observed in 30% of patients when treated with BAT, and 52% had a PSA response when retreated with enzalutamide. In the abiraterone cohort, PSA50 was 17% and only 16% had PSA response in retreatment [18, 19]. Finally, in the report of the cohort examining BAT as a first-line hormonal treatment for metastatic CRPC (patients not exposed to AR-targeted therapies), BAT was well tolerated and resulted in prolonged disease stabilisation, with favourable responses to subsequent second-generation AR-targeted therapies [20].

Several limitations of this analysis should be addressed. Small number of patients and a retrospective, non-randomised study limit the interpretation of data. Also, the heterogeneous population included and without a uniform indication for BAT. Even though, we observed a similar RR with enzalutamide re-challenge as in the Teply *et al* [18] trial and a slightly shorter PFS2 compared to Denmeade *et al* [17] trial. These findings support the hypothesis that BAT can re-sensitise prostate cancer cells to enzalutamide, even after several lines of treatment, and raise the question to whether BAT should be offered to these patients. mCRPC patients have limited options after becoming resistant to new hormonal agents. Usually, we use chemotherapy and/or radiopharmaceutical agents such as radium-223 or ^177^Lu-PSMA. These treatments should be offered to our patients because they have showed survival advantage in randomised phase 3 clinical trials, but in many instances, patients progress to all these available treatments and had no other options, been BAT the option.

In Latin America countries as well in lower or lower-middle income countries, access to this newer treatment is not always universal so we have to consider lines of treatment that are accessible in matter of costs and taking into account improvement of quality of life and prolonging hormonal sensitivity.

Among the advantages of using BAT, is the good tolerance to treatment, and this makes its combination with different agents such as new hormonal agents or immunotherapy feasible. To highlight, we mention the study by Markowski *et al* [31] where patients who received BAT and were then exposed to immunotherapy experienced 100% decreases in PSA and one patient remained in long-term remission turning this strategy into a hypothesis to be studied in future trials.

## Conclusion

These results were not enough to change practice in these institutions, but more physicians seem more prone to its use, especially for patients in which the effects of androgen deprivation greatly affect their quality of life, or for patients not suitable for chemotherapy. We encourage medical oncologist to discuss all these patients into a multidisciplinary tumour board.

There is still much to learn about this strategy, and more efforts should be put in prospective trials to test these findings. Until now, the only clear criterion to use BAT is in asymptomatic patients (due to the probability of worsening pain secondary to a flare effect), and perhaps a more detailed evaluation of biomarkers could help to better select patients. In addition, we believe that quality of life and cost-effectiveness studies are also necessary (even though this was not tested in our study), since it seems that these would be the greatest benefits of this therapy.

## Conflicts of interest

None of the authors have conflicts of interest to declare.

## Funding

The authors have not declared a specific grant for this research from any funding agency in the public, commercial or not-for-profit sectors.

## Figures and Tables

**Table 1.** Characteristics of the patients at baseline.

Characteristic	*N* (%): 21
Median age (range), years	75 (64–83)
Gleason score • ≤7 • ≥8 • Unknown	7 (33.3%) 4 (19%) 10 (47.6%)
HRR testing • Somatic • Germline • Both • None	4 (19%) 4 (19%) 3 (14.2%) 10 (47.6%)
HRR mutations • Yes • No • Unknown	0 11 (52.38%) 10 (47.6%)
Number of previous lines • Median (range)	2 (1–5)
Previous treatments • NHA • Abiraterone • Enzalutamide • Chemotherapy • Radium-223 • Others	21 (100%) 7 (33.3%) 15 (71.4%) 13 (61.9%) 10 (47.6%) 1 (4.8%)
PSA previous to BAT • Median ng/mL (range)	75 (0.06–1386)
Baseline symptoms • Asymptomatic • Mild pain	19 (90.47%) 2 (9.52%)

HRR, Homologous recombination repair

**Table 2.** Summary of cases.

Patient	Age	Number of previous lines	PSA before BAT	*n*° cycles of BAT	Best response	Next treatment	Response to next treatment	PFS1 days (Fup)	PFS2 days (Fup)
1	71	2	90	4	PSA50	Radium	PD	124	201
2	81	1	26.72	2	PD	Docetaxel	PR	64	253
3	83	1	6	2	PD	-	-	62	-
4	83	1	20	8	PR and PSA50	Enzalutamide	PR	239	414
5	64	3	20.9	2	PD	Enzalutamide	PD	56	112
6	74	4	416	5 (ongoing)	SD	-	-	NR (159)	-
7	79	2	84.66	4	PR	Carboplatin	-	119	NR (143)
8	79	5	928	2	PD	Carboplatin	-	70	139
9	76	1	5.85	5	SD	Enzalutamide	SD	139	238
10	72	3	39.3	3	PD	Enzalutamide	PR and PSA50	92	216
11	79	2	254	3	SD	Docetaxel	PR and PSA50	105	345
12	71	3	238.96	3	PSA50	Enzalutamide		101	NR (101)
13	78	2	1,386	1 (ongoing)	NR	-	-	NR (13)	-
14	72	2	22.8	1 (ongoing)	NR	-	-	NR (15)	-
15	71	3	334	2	PSA50	Abiraterone	PD	70	114
16	75	3	0.06	17	PR and PSA50	Abiraterone	PR and PSA50	485	606
17	76	1	NR	44	SD	Enzalutamide	PR and PSA50	1,250	1,684
18	71	3	21	3	PSA50	Enzalutamide	PSA50	101	NR (101)
19	71	3	127	4	SD	Lu-PSMA	PD	139	202
20	76	2	43.3	2	PD	Docetaxel	-	62	NR (62)
21	79	3	75	14	SD	-	-	401	462

PSA, Prostate specific antigen; BAT, Bipolar androgen therapy; PFS, Progression free survival; PD, Progressive disease; SD, Stable disease; PR, Partial response; PSA50, 50% decrease in prostate specific antigen; NR, Not reported
